# Diagnostic Value of Serum Periostin for Cyst Involution in Children with Multicystic Dysplastic Kidney

**DOI:** 10.3390/jcm14207264

**Published:** 2025-10-15

**Authors:** Agnieszka Szmigielska, Iwona Kotuła, Urszula Demkow, Maria A. Szmigielska, Agnieszka Tutka

**Affiliations:** 1 Department of Pediatrics and Nephrology, Medical University of Warsaw, 02-091 Warsaw, Poland; agnieszka.tutka@uckwum.pl; 2 Department of Laboratory Diagnostics and Clinical Immunology of Developmental Age, Medical University of Warsaw, 02-091 Warsaw, Poland; iwona.kotula@wum.edu.pl (I.K.); urszula.demkow@wum.edu.pl (U.D.); 3 Student Scientific Group, Department of Pediatrics and Nephrology, Medical University of Warsaw, 02-091 Warsaw, Poland; s088469@student.wum.edu.pl

**Keywords:** multicystic dysplastic kidney, periostin, renal cysts, kidney dysplasia

## Abstract

**Background:** In polycystic kidney diseases, increased periostin levels and disease progression are observed. Multicystic dysplastic kidney (MCDK) is characterized by kidney atrophy. The aim of the study is to assess serum periostin activity in children with MCDK and in patients with MCDK and involution of cysts. **Methods:** We enrolled in the study 64 children aged 1–5 years (mean age 25 +/− 9 months). Serum periostin (sPOSTN) levels were measured using enzyme-linked immunosorbent assay. We divided children into three groups: group A—patients with MCDK and cysts (*n* = 34); group B—patients with involution of cysts or after nephrectomy (*n* = 10), and group C—healthy children (*n* = 20). **Results:** Blood samples were obtained from 64 children, including 44 children with MCDK (26 boys, 18 girls). sPOSTN levels were significantly higher in group A, 239.1 ± 168.1 [IQR: 62.4, 385.7] ng/mL, compared to group B, 77.7 ± 82.8 [IQR: 31.7, 117.0] ng/mL (*p* < 0.05). The median level of periostin in patients with MCDK (groups A and B) was 204.4 ± 168.2 [IQR: 34.9, 363.4] ng/mL and 141.1 ± 129.0 [IQR: 30.3, 276.9] ng/mL in group C, respectively. In patients with the renal cysts, the cut-off value of periostin was 133.57 ng/mL. The lowest level of periostin was observed in patients in group B. There were no significant differences in periostin level between groups B and C. **Conclusions:** The study shows that a high level of sPOSTN was identified in patients with MCDK and cyst presentation in abdominal ultrasonography. The level of sPOSTN could be a promising blood marker of the cyst’s formation in cystic kidney diseases. However, this study remains preliminary; further studies are needed to confirm our findings.

## 1. Introduction

The renal cystic diseases are a group of disorders characterized by the formation of cysts in the kidneys. They include varied and important clinical entities from the simple renal cysts to autosomal dominant polycystic kidney disease (ADPKD), autosomal recessive polycystic kidney disease (ARPKD), medullary sponge kidney (MSK), multicystic dysplastic kidney (MCDK), and acquired cystic kidney disease (ACKD) [1]. The incidence varies worldwide depending on the specific type of renal cystic disease. In the adult population, simple renal cysts are detected in approximately 25–33% of patients over 50 years of age. Other forms of cystic disease can affect patients with the following frequencies: ADPKD 1:500–1:1000, ARPKD 1:20,000, MSK 1:5000–1:20,000, and MCDK 1:4300 [2,3,4,5]. ACKD is common in patients with chronic kidney disease, and its prevalence increases with the duration of dialysis [6]. In most cystic renal diseases (ADPKD, ARPKD, MSK, ACKD), a systematic increase in the number of cysts and progression to chronic or end-stage renal disease is often observed. However, MCDK is the exception among cystic diseases. It is a relatively common renal defect in children and is usually unilateral. The condition is more common in males and tends to affect the left kidney more frequently. An infant with multicystic dysplastic kidney (MCDK) is born with a dysplastic kidney containing numerous cysts of varying sizes that replace the normal renal parenchyma. This kidney is non-functional and usually undergoes involution during the first 2 years of the child’s life. However, most patients with unilateral MCDK lead normal lives with good renal function secured by the unaffected kidney. But potential complications include hypertension, urinary tract infections, and malignant transformation within the dysplastic kidney. Despite common presentation in humans, the cause of kidney atrophy in MCDK is unknown [7,8,9,10,11].

The hypothesis of our study is that periostin can be one of the markers of cyst progression in kidneys. Periostin (90 kDa) is encoded by the osteoblast-specific factor-2 gene. It belongs to the matricellular proteins (MPs) and directly interacts with the extracellular matrix (ECM). Periostin activates pathways like integrin-linked kinase (ILK) and focal adhesion kinase (FAK). ILK and FAK can influence cell proliferation and cyst enlargement. Normal expression of periostin is well-described during embryogenesis in periosteum, tendons and ligaments, heart, lungs, skin, and teeth [12,13]. In adults, periostin expression increases in areas undergoing significant mechanical stress, injury, or pathological changes, especially in diseases with inflammation and neoplastic growth. Its high expression is seen in neoplasms of the brain, ovary, liver, and lung, but also in asthma, ADPKD, and in patients after renal transplantation [14,15,16,17,18,19].

The aim of our research was to assess the concentration of serum periostin (sPOST) in children with MCDK and the presence of cysts on ultrasound examination and to compare them to children with MCKD and cyst involution, as well as to healthy children as a control group. Furthermore, we would like to establish sPOST as the cut-off value between children with active and inactive cyst formation in the kidney.

## 2. Materials and Methods

This prospective observational single-center study was performed in children with MCDK diagnosed in the Department of Pediatrics and Nephrology at the Medical University of Warsaw. We enrolled in the study 44 patients with MCDK aged 12–60 months (mean age 25 +/− 9 months). The control group consisted of 20 healthy children adjusted for the same age. The patients’ standard clinical parameters were assessed by measuring temperature, weight, height, and blood pressure. Children with urinary tract infection (UTI) or any other infections were excluded from the study. In all children, blood tests including white blood cell count (WBC), C-reactive protein (CRP), serum concentration of creatinine (Cr) and urea (Ur), and microalbuminuria, were performed. The normal values of the tests were set according to the local reference laboratory as follows: CRP ≤ 1.0 mg/dL, Cr ≤ 0.4 mg/dL, Ur 15–30 mg/dL, and WBC according to the age. The blood test was performed using the VITROS 5600 Integrated System, Ortho Clinical Diagnostics (San Diego, CA, USA). Based on the abdominal ultrasonography (US), we divided children into three groups: group A—patients with MCDK and cysts (*n* = 34); group B—patients with cyst involution or after nephrectomy (*n* = 10); and group C—healthy children (*n* = 20) with normal kidneys. Group B included 6 children with cyst involution and 4 children after nephrectomy due to significant large MCDK. Cyst involution was defined as complete ultrasonographic disappearance of macroscopic cysts with residual atrophic renal tissue or complete loss of identifiable renal parenchyma in the renal fossa in patients post-nephrectomy. Healthy controls (group C) were recruited from children undergoing routine well-child evaluations or minor elective procedures, with no history of renal disease or systemic illness.

In all children, serum concentration of periostin (sPOST) was checked. A serum sample for the measurement of sPOST was collected in the morning, then it was immediately centrifuged and stored at −80 °C until further evaluation. The levels of sPOST were determined using commercially available enzyme-linked immunosorbent assay (ELISA kit, catalog No.201-12-4519, SunRed Biological Company, Shanghai, China) according to the protocol provided by the manufacturer. The study was approved by the Bioethical Committee of the Medical University of Warsaw, Poland (KB/48/2016), and was conducted in accordance with the guideline of the Declaration of Helsinki (2013). For the statistical analysis, we used program Statistica 13.3 PL software (StatSoft, Tulsa, OK, USA). Data are presented as incidences, percentages, mean and standard deviation (SD), or median and interquartile ranges (IQRs). For normally or non-normally distributed variables, Student’s *t*-test or the Mann–Whitney U-test were performed, respectively. The Kruskal–Wallis test was used to compare the level of periostin in three groups. Receiver operating curve (ROC) analysis was performed to establish the cut-off, specificity, and sensitivity of sPOST. *p* values less than 0.05 were considered statistically significant.

## 3. Results

We evaluated 44 children with MCDK (26 boys, 18 girls) and 20 (10 boys, 10 girls) healthy children. The clinical characteristics and laboratory data of the 64 study patients divided into three groups (A-MCDK/cyst+, B-MCDK/cyst−, C-control) are shown in Table 1. Normal body weight and height of patients in all groups ruled out malnutrition. Kidney function parameters, microalbuminuria, and blood pressure were normal in all patients from three groups. Normal CRP and leukocytosis excluded potential significant infection.

The highest mean level of sPOST (239.1 ng/mL) was noticed in the group A, and the lowest mean level of sPOST (77.7 ng/mL) in group B. A comparison of periostin level (ng/mL) as a mean value, median, ranges, and standard deviation in children from groups A, B, and C is presented in Table 2.

**Figure 1:**
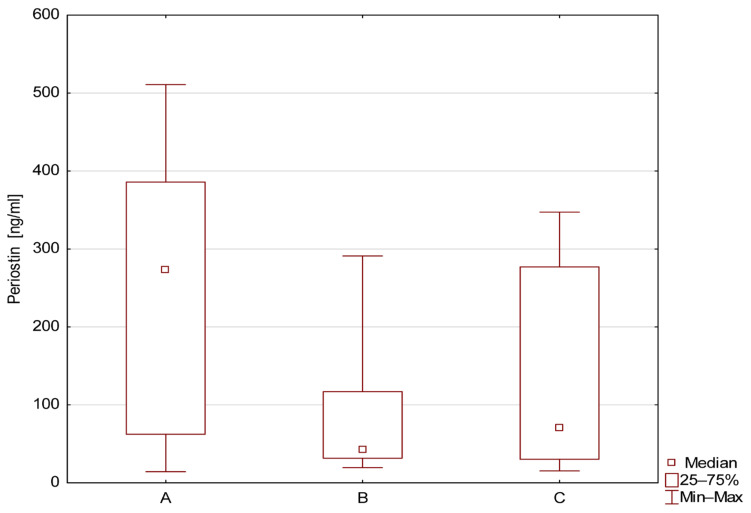
Level of serum periostin (ng/mL) in patients from groups A, B, and C.

The mean level of sPOST was significantly higher and was statistically significant (*p* < 0.05) in children with MCDK and cysts compared to children from group B (solitary kidney). The post hoc test confirmed a significantly higher level of sPOST in group A. There were no significant differences between groups B and C, as shown in Figure 1.

**Figure 2:**
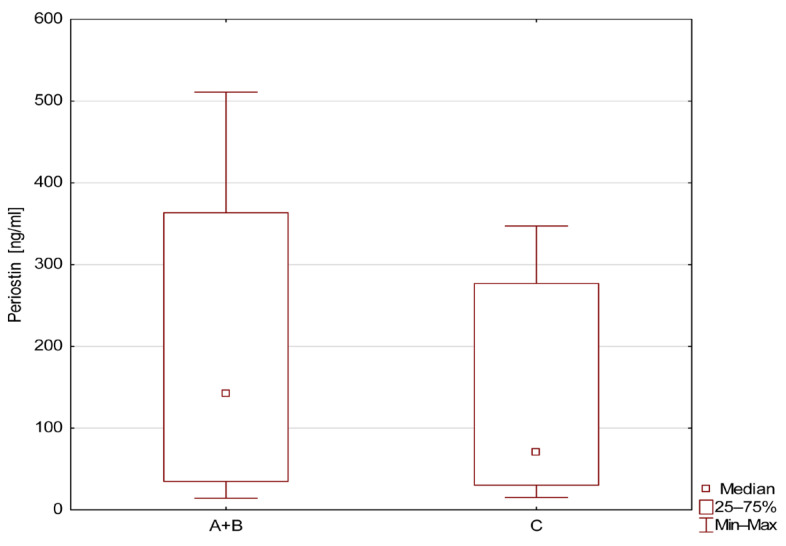
Level of serum periostin (ng/mL) in patients with MCDK (with and without cysts, A+B) and healthy controls.

Children with MCDK with cysts and with involution or after nephrectomy had higher levels of sPOST (A+B group) compared to healthy children, as shown in Figure 2.

The ROC analysis showed the highest usefulness of sPOST in group A for detecting active processes of cyst involution. A comparison of A and B groups revealed high specificity (88.9%) for detection of cysts in patients with MCDK, as shown in Table 3.

**Figure 3:**
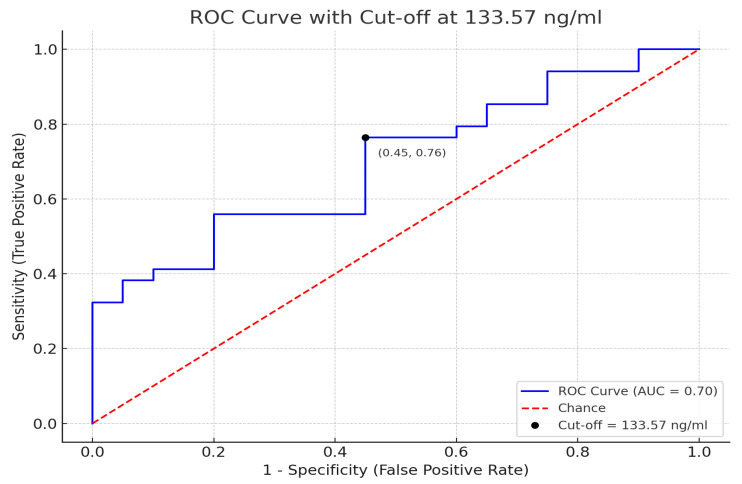
Specificity and sensitivity were calculated for a cut-off of 133.57 ng/mL.

The ROC analysis yielded an AUC of 0.81 (95% CI: 0.69–0.93), and the optimal cut-off for distinguishing patients with cystic presentation (group A) from those with involution/post-nephrectomy (group B) was 133.57 ng/mL (95% CI: 121.4–145.2), as shown in Figure 3.

No significant correlations were found between serum periostin and creatinine (r = 0.12, *p* = 0.34), urea (r = 0.09, *p* = 0.48), or microalbuminuria (r = 0.15, *p* = 0.28). This may reflect preserved overall renal function.

## 4. Discussion

Multicystic dysplastic kidney (MCDK) represents a form of renal dysplasia arising from abnormal renal morphogenesis. Among the genes implicated in its development, TGF-β and PAX2 play central roles, as they regulate expression of uroplakin, a structural protein essential for maintaining the integrity and impermeability of the urinary tract epithelium [20]. Approximately 20% of congenital kidney malformations have a monogenic origin, and 4% may result from copy number variants (CNVs) [21,22]. Abnormal embryogenesis activates renal repair mechanisms that may persist postnatally, contributing to continued dysplastic remodeling.

Periostin, first identified in 1993 [23], is a matricellular protein involved in diverse physiological processes, including embryonic development, extracellular matrix (ECM) organization, bone remodeling, and odontogenesis. Its expression is regulated by TGF-β, and the protein is particularly abundant in tissues rich in collagen, such as cardiac valves and tendons [24]. In healthy individuals, serum periostin concentrations remain low, reflecting its limited expression in mature, quiescent tissues. In contrast, periostin levels increase in response to tissue injury, irrespective of etiology, and are elevated in conditions associated with fibrosis, inflammation, or neoplasia. In renal pathology, chronic injury leads to progressive fibrosis mediated by multiple pathways, including angiotensin II, TGF-β, endothelin-1, epidermal growth factor, and platelet-derived growth factor, all of which stimulate ECM deposition and fibroblast activation [25,26]. Periostin is one of the most highly expressed proteins in human autosomal dominant polycystic kidney disease (ADPKD), where it correlates with cyst expansion and fibrotic progression [27].

In contrast to ADPKD, MCDK typically undergoes spontaneous involution during the first five years of life [28,29]. In this context, our study focused on periostin concentrations in children aged 1–5 years with MCDK, demonstrating significantly elevated serum periostin (239.1 ng/mL) in patients with cystic kidneys compared to 77.7 ng/mL in children with cyst involution or post-nephrectomy. The reduction in periostin levels following cyst regression suggests a loss of active fibrotic remodeling rather than a direct suppression of periostin synthesis. Indeed, children with complete cyst involution or surgical removal of the dysplastic kidney (group B) likely exhibit reduced renal parenchymal mass and diminished periostin production. Interestingly, healthy controls (group C) showed moderately higher periostin levels than group B, possibly reflecting baseline developmental expression of periostin during early childhood, when it contributes to growth and tissue maturation. Elevated periostin levels in children with persistent renal cysts likely indicate ongoing reparative and fibrotic processes within dysplastic parenchyma. As fibrosis progresses, cyst involution and renal atrophy ensue. In children with unilateral MCDK, functional compensation depends on the contralateral hypertrophied kidney. Although compensatory hypertrophy ensures adequate renal function, the total nephron number remains lower than in children with two anatomically normal kidneys. Notably, in our cohort, involution occurred despite elevated periostin, suggesting that periostin may not be the direct driver of cyst persistence but rather a marker of tissue remodeling. The mechanisms governing cyst regression in MCDK remain incompletely understood, and the regulation of periostin in this process warrants further investigation. Our cross-sectional design limits assessment of temporal changes in periostin levels during cyst evolution. Future longitudinal studies, incorporating serial periostin measurements and ultrasound-based monitoring of cyst involution, are necessary to delineate periostin’s temporal dynamics and prognostic value.

In ADPKD, persistently elevated periostin parallels progressive cyst growth, whereas in MCDK, periostin is also elevated but associated with an opposite clinical course—cyst involution and renal atrophy. Thus, serum periostin may serve as a stage-specific biomarker, reflecting active cystic remodeling rather than inexorable progression. The ROC-derived cut-off (133.57 ng/mL) distinguished active cystic disease (group A) from resolved or absent cysts (group B) with high sensitivity and specificity. Although this threshold lies below the mean periostin level observed in healthy controls, this discrepancy likely reflects developmental periostin expression in normal tissues unrelated to cystic pathology. Importantly, the ROC analysis was designed for intra-cohort discrimination, not for differentiating MCDK from healthy individuals.

Clinically, serum periostin may aid in identifying ongoing cystic activity and guiding follow-up strategies. In most children, MCDK involutes by five years of age, rendering the dysplastic kidney ultrasonographically undetectable. However, in cases with persistent cystic remnants beyond 10 years, the kidney typically appears small and scarred, necessitating continued surveillance to confirm complete atrophy [30,31,32]. Our study, encompassing 44 patients with MCDK from a single tertiary center, has several limitations. The small sample size, particularly in group B (*n* = 10), may limit statistical power and the detection of subtle associations. Recruitment of children post-nephrectomy remains challenging, as nephrectomy rates have declined significantly with recognition of natural cyst involution. Historically, prophylactic nephrectomy was performed due to oncologic concerns, but current evidence supports a conservative approach, with nephrectomy rates now approximately 8–13% [33,34,35,36]. In our cohort, only 9% underwent surgical removal. Additionally, the control group was not sex-matched, which may influence periostin levels given sex-dependent differences in early growth and ECM turnover. Future studies should therefore implement age- and sex-matching to minimize confounding. Mechanistically, both ADPKD and MCDK share features of increased intracystic pressure and mechanical stress on renal parenchyma, which activate repair signaling pathways. In ADPKD, these pathways become maladaptive, promoting relentless cyst expansion and fibrosis. In contrast, in MCDK, similar stimuli may lead to successful tissue remodeling and cyst involution. Thus, periostin may function as a context-dependent regulator: persistently high levels, as seen in ADPKD, may perpetuate fibrotic remodeling and cyst proliferation, whereas transient expression in MCDK may facilitate repair and eventual regression. Future multicenter studies with larger, balanced cohorts and serial sampling are needed to confirm periostin’s role in this differential remodeling process.

## 5. Conclusions

In summary, this study demonstrates high periostin expression in children with MCDK and cystic presentation on ultrasonography. In contrast, periostin concentrations were low in patients with cyst involution or after nephrectomy. These findings suggest that serum periostin reflects active cystic remodeling and declines as cysts regress. This unique pattern positions periostin as a potential biomarker of cyst activity and involution in cystic renal diseases. However, this study remains preliminary; further studies are needed to confirm our findings.

## Figures and Tables

**Table 1:** Baseline clinical and laboratory data in the study and control groups.

	Group A *N* = 34 (53.1%)	Group B *N* = 10 (15.6%)	Group C *N* = 20 (31.3%)	*p* Value
Age (months)	36 (9)	38 (6)	34 (7)	NS
Boys/Girls	21/13	5/5	10/10	NS
Height (cm)	95 (7)	90 (4)	90 (6)	NS
Weight (kg)	15 (4)	14 (5)	15 (6)	NS
BP (mmH) according to age	Normal	Normal	Normal	NS
CRP (mg/dL)	<0.5	<0.5	<0.5	NS
White blood cells (×10^9^/L)	7.86	8.28	6.34	NS
Urea (mg/dL)	23	16	15	NS
Creatinine (mg/dL)	0.31	0.39	0.41	NS

Group A = MCDK/cyst+, group B = MCDK/cyst−, and group C = control group. NS = not significant. CRP, WBC, urea, creatinine—mean value.

**Table 2:** Comparison of periostin level (ng/mL) in children from groups A, B, and C.

	Number of Patients	Mean ± SD	Median	Min	Max	QR1	QR3
A	34	239.1 ± 168.1	273.3	14.3	510.9	62.4	385.7
B	10	77.7 ± 82.8	42.4	19.5	290.9	31.7	117.0
C	20	141.1 ± 129	70.8	15.3	347.3	30.3	276.9

**Table 3:** ROC analysis in study patients.

Group	AUC	95%CI Upper–Lower	SE	z	Sensitivity %	Specificity %	*p*
A vs. C	0.7	0.527–0.813	0.073	2.342	76.5	50	<0.05
B vs. C	0.608	0.405–0.810	0.103	1.040	-	-	0.298
A vs. B	0.765	0.618–0.912	0.075	3.530	67.6	88.9	<0.05
A+B vs. C	0.609	0.467–0.750	0.072	1.504	-	-	0.132

## Data Availability

The original contributions presented in this study are included in the article. Further inquiries can be directed to the corresponding author.
